# A Treatise on Venereal Disease

**Published:** 1859-03

**Authors:** 


ART. X —*A Treatise on Venereal Disease.* By JOHN HUNTER, F.R.S., with Copious Additions, by Dr. PHILIP RICORD. Translated and edited, with Notes, by FREEMAN J. BUMSTEAD, M.D., Lecturer on Venereal at the College of Physicians and Surgeons, New York, etc. Second Edition, revised, containing a Résumé of Ricord's Recent Lectures on Chancre. 8vo., pp. 552. Blanchard & Lea: Philadelphia. 1859.

THE present edition of Ricord and Hunter on venereal is rendered more interesting and complete by the additions of the American editor, of the recent views of Dr. Ricord on chancre, of which he has incorporated full notes in the text. A complete summary of these views will be found in the second volume of this journal, in a review of M. Fournier's work, so that it will be unnecessary to speak of them at this time. This work has been for a number of years before the profession, and has met with their warmest approval, its teachings being almost universally adopted. We give it our most hearty commendations, believing, as we do, that it is the most able, satisfactory, and comprehensive work of the kind, on the subject of which it treats, in any language.

